# How correlations between treatment access and surveillance inclusion impact neglected tropical disease monitoring and evaluation—A simulated study

**DOI:** 10.1371/journal.pntd.0011582

**Published:** 2023-09-06

**Authors:** Jessica Clark, Emma L. Davis, Joaquin M. Prada, Katherine Gass, Alison Krentel, T. Déirdre Hollingsworth

**Affiliations:** 1 School of Biodiversity, One Health & Veterinary Medicine, University of Glasgow, Glasgow, Scotland; 2 Big Data Institute, Neglected Tropical Disease Modelling Consortium, University of Oxford, Oxford, England; 3 School of Veterinary Medicine, Faculty of Health and Medical Sciences, University of Surrey, Guildford, England; 4 Neglected Tropical Diseases Support Center, Task Force for Global Health, Decatur, Georgia, United States of America; 5 School of Epidemiology and Public Health, University of Ottawa, Ottawa, Canada; 6 Bruyère Research Institute, Ottawa, Canada; The University of Sydney School of Veterinary Science, AUSTRALIA

## Abstract

Neglected tropical diseases (NTDs) largely impact marginalised communities living in tropical and subtropical regions. Mass drug administration is the leading intervention method for five NTDs; however, it is known that there is lack of access to treatment for some populations and demographic groups. It is also likely that those individuals without access to treatment are excluded from surveillance. It is important to consider the impacts of this on the overall success, and monitoring and evaluation (M&E) of intervention programmes. We use a detailed individual-based model of the infection dynamics of lymphatic filariasis to investigate the impact of excluded, untreated, and therefore unobserved groups on the true versus observed infection dynamics and subsequent intervention success. We simulate surveillance in four groups–the whole population eligible to receive treatment, the whole eligible population with access to treatment, the TAS focus of six- and seven-year-olds, and finally in >20-year-olds. We show that the surveillance group under observation has a significant impact on perceived dynamics. Exclusion to treatment and surveillance negatively impacts the probability of reaching public health goals, though in populations that do reach these goals there are no signals to indicate excluded groups. Increasingly restricted surveillance groups over-estimate the efficacy of MDA. The presence of non-treated groups cannot be inferred when surveillance is only occurring in the group receiving treatment.

## Author summary

Mass drug administration (MDA) is the cornerstone of control for many neglected tropical diseases. As we move towards increasingly ambitious public heath targets, it is critical to investigate ways in which MDA weaknesses can be strengthened. It is known that some individuals systematically choose not to participate in treatment. It is also becoming evident that others systematically do not have access to treatment. What is less clear however, is how access to treatment correlates to inclusion in surveillance efforts, and in turn, how this impacts the monitoring and evaluation of intervention programmes. If individuals with access to treatment are more likely to be included in surveillance efforts, then this implies that those without access to treatment are likewise more likely to be excluded from surveillance. Extending the individual-based lymphatic filariasis model, TRANSFIL, we show that exclusion to treatment and surveillance negatively impacts the probability of reaching public health goals, though in populations that do reach these goals there are no signals to indicate excluded groups. Increasingly restricted surveillance groups over-estimate the efficacy of MDA. The presence of non-treated groups cannot be inferred when surveillance is only occurring in the group receiving treatment.

## Introduction

Neglected tropical diseases (NTDs) are a heterogeneous group of diseases listed by the World Health Organization (WHO) as causes of great human, social and economic burden, largely impacting vulnerable marginalised communities in tropical and subtropical regions [1]. Reducing the burden of infection for some of these diseases has been achieved through mass drug administration (MDA) which refers to the distribution of medicine to an entire eligible population or specific subset (for example, those over a certain age) within a given administrative area regardless of individual infection status [1]. It is widely accepted that the treatments chosen for MDA have a good safety profile, such that there is little risk from treating uninfected people. In addition, test and treat methods can be resource costly, where MDA can reduce costs significantly. This is particularly relevant for high prevalence diseases/ locations and for diseases where diagnosis is more difficult or invasive (for example onchocerciasis where skin snip biopsies must be conducted, often in multiples to increase diagnostic sensitivity [2]). Based on estimates of prevalence before and/or during a programme within administrative units, the WHO provides recommended treatment frequencies and coverage levels. These have recently been updated for the next decade in the 2021–2030 roadmap [1]. Unfortunately, for the NTDs where MDA is the cornerstone of control, achieving recommended coverage levels remains an ongoing challenge that negatively impacts progress towards elimination as a public health problem (EPHP) [1,3].

Sub-optimal treatment coverage can be due to a lack of access to treatment opportunities, or a lack of adherence to treatment regimens. Recent efforts have been made to unify the terminology used by the global community, with `never treatment`covering both routes of missed or never received treatment [3]. For the purposes of this analysis, we need to delineate the two so that we can be clear on which types of behaviour we are representing in the model. A lack of treatment access (exclusion) can occur for many reasons, including impractical timing of treatment delivery (for example during the day for those away at work), because community treatment distributors do not go to certain homes or areas, or because people themselves cannot access facilities where treatments are distributed from [4,5]. We refer to this as systematic non-access and the group without access as being excluded. It is also possible that inclusion in surveillance efforts is correlated to treatment access, such as with rabies [6], schistosomiasis [4] and lymphatic filariasis [7]. In this instance, people with poor access may be excluded from both treatment and surveillance efforts—influencing estimates of prevalence, and subsequent programme decision making. For several NTDs, modelling has shown that sufficient treatment coverage should suppress transmission to a breakpoint [8,9] below which transmission is no longer sustainable [10]. However, if a sufficiently large sub-population are not receiving treatment and are not accounted for in surveillance efforts but are nonetheless contributing to transmission, target breakpoints may not be reached, or may be perceived as reached but not maintained–driven by this undetected reservoir of infection. It is therefore critical to understand the impact of such groups and whether the level of exclusion can be inferred.

The other commonly cited reason for lower than target coverage, is non-adherence. In this work, this refers to the successful delivery and distribution of treatment to diverse populations, but an active decision by an individual to not participate/ ingest medication or use it as intended [11–14]. This is in contrast to matters of treatment access, which is not a choice made by the recipient. Where this non-adherence choice is made repeatedly over successive rounds of MDA, we term this systematic non-adherence. Reasons for this behaviour are numerous, including (but not limited to) fear of side effects [15,16], belief systems [17] and misinformation regarding safety (for example, schistosomiasis treatment with praziquantel during pregnancy)[18,19]. Quantifying the proportion of the population this pertains to is somewhat possible through behavioural questionnaires giving better insight into who is actually being treated [15,20]. Evidence suggests that treatment registers are often incomplete highlighting the need for other methods to detect ‘never treated’ individuals [21], which is ultimately vital to accurately estimating the efficacy of intervention programmes based largely on MDA.

Of the five diseases that rely on MDA for control, the WHO identified improving access and delivery as a critical action point for lymphatic filariasis. As a case study, we extend the highly detailed individual-based model, TRANSFIL [22,23], simulating the infection dynamics of lymphatic filariasis at the level of the individual human host. We use this model to investigate the impact of unmeasured, untreated, and therefore unobservable groups, on “true” versus observed (i.e., measurable by surveillance) infection prevalence in humans. Using simulated populations with a range of baseline prevalences, we assessed the probability of reaching EPHP targets as a function of exclusion proportions. We also provide estimates of the number of rounds of treatment needed to reach these targets in the presence of exclusion. Finally, we investigated whether it was possible to relate surveillance insight to the exclusion proportion. We simulated the transmission assessment survey (TAS), conducted after at least five years of effective treatment [24], which is used by lymphatic filariasis programmes to determine whether MDA can be stopped. As the first evaluation survey required of lymphatic filariasis programmes, this is the time point at which challenges in achieving the goals are most frequently detected [25].

## Methods

The TRANSFIL model framework has been described in detail and validated in various populations and settings elsewhere [23]. Parameter values are given in supplementary material, where a link to all code can also be found. In short, in this stochastic model, the male and female worm burdens within each human host are modelled, with fertile female worms producing microfilaria (mf), and with the density of infectious third stage larvae (L3) in the human-biting mosquito population reflecting the overall microfilaraemia density in the human population and the infection pressure. Demographic characteristics of each human host are modelled including age, sex and exposure risk. Here, we present simulations relevant to most of Africa where we consider *Anopheles-*driven transmission and treatment with Ivermectin and Albendazole. We do not simulate *Culex*-driven transmission that simulates India-like populations where treatment is with Diethylcarbamazine and Albendazole treatment, however, we expect qualitatively similar results with respect to exclusion.

### Simulated populations

We simulated 35,000 populations with baseline prevalences from 0 to ~60% (Fig A in S1 Supplementary Material). Prevalence was modulated via the vector-to-host ratio and the bite rate aggregation parameters (*k)* with increasing prevalence associated with higher vector-to-host ratios and more randomly distributed bites (Fig B in S1 Supplementary Material; parameters in Table A in S1 Supplementary Material). Populations were filtered to have a minimum of baseline 1% prevalence across the population over the age of five. There were a total of 14,145 populations used in the simulations. The same populations were reset to their baseline state and used in each of the diagnostic/ access scenarios.

### Simulated diagnostics

To determine the impact of non-access on prevalence estimates and treatment efficacy we observed prevalence in four groups, using the two main diagnostic methods available. When using the filariasis test trip (FTS) diagnostic, the operational target prevalence that indicates elimination as a public health problem is 2%. When using detectable mf prevalence from bloody smears (or similar), the EPHP indicated prevalence is 1%

FTS diagnostic: We simulated FTS diagnostic use in three groups with target EPHP prevalence as above; 1) the whole population > five-years-old (referred to as True Prevalence), 2) six- and seven-year-olds (referred to as TAS, see ref [24]), 3) in those aged >five-years-old and with access (Community-wide with access). We assumed male or female worm presence would be detected with a 93.1% sensitivity [26] without considering false positives from coinfection.

Blood smear (or similar): Based on expert advice from co-author Dr Katherine Gass, as has been discussed in decision-making settings, we simulated detectable mf prevalence in those >20-year-olds (referred to as mf TAS).

### Simulated treatment

To investigate the probability that five years of treatment would result in achieving the desired threshold, after 100 years burn-in populations received treatment for five years, with an additional treatment at the sixth year, as is common due to the timing of the pre-TAS [24]. We explored the dynamics under no exclusion (where exclusion refers to treatment access and inclusion in surveillance), 10% exclusion, and 50% exclusion (model methods below). Whilst 50% exclusion may seem high, evidence from the lymphatic filariasis literature suggests that treatment repeatedly failed to reach certain homes, resulting in a large percentage of treatment non-access [11,15,17,27]. This problem is not confined to lymphatic filariasis (e.g., schistosomiasis [4,5]), and given the translatability of these ideas to other models, this justifies the investigation into the impacts of such a high exclusion percentage.

To investigate the dynamics once treatment stopped when the appropriate thresholds were met, we recorded prevalence in our observed groups every month for 12 years. We present this part of our analysis focussing on the populations that reached the threshold targets by the fifth MDA. To investigate the number of rounds of treatment needed to reach the operational targets as a function of baseline prevalence and the exclusion proportion in our observed populations, treatment was continued until the target was reached or the simulation was allowed to continue for 40 years at which point the simulation would stop and note the failure to reach the target.

### Surveillance inclusion, treatment access, & adherence

The model framework was adapted to investigate the impact of a correlation between treatment access, and inclusion in surveillance. All populations were targeted with 65% treatment coverage that was applied to the observable surveillance groups with access. Treatment access and inclusion in surveillance had a far stronger impact on infection dynamics than adherence (supplementary material Fig C in S1 Supplementary Material). We therefore focus on the impact of increasing exclusion in the results. These and all subsequent investigations used a moderate level of non-adherence (0.2) in line with previous investigations [28].

**Fig 1:**
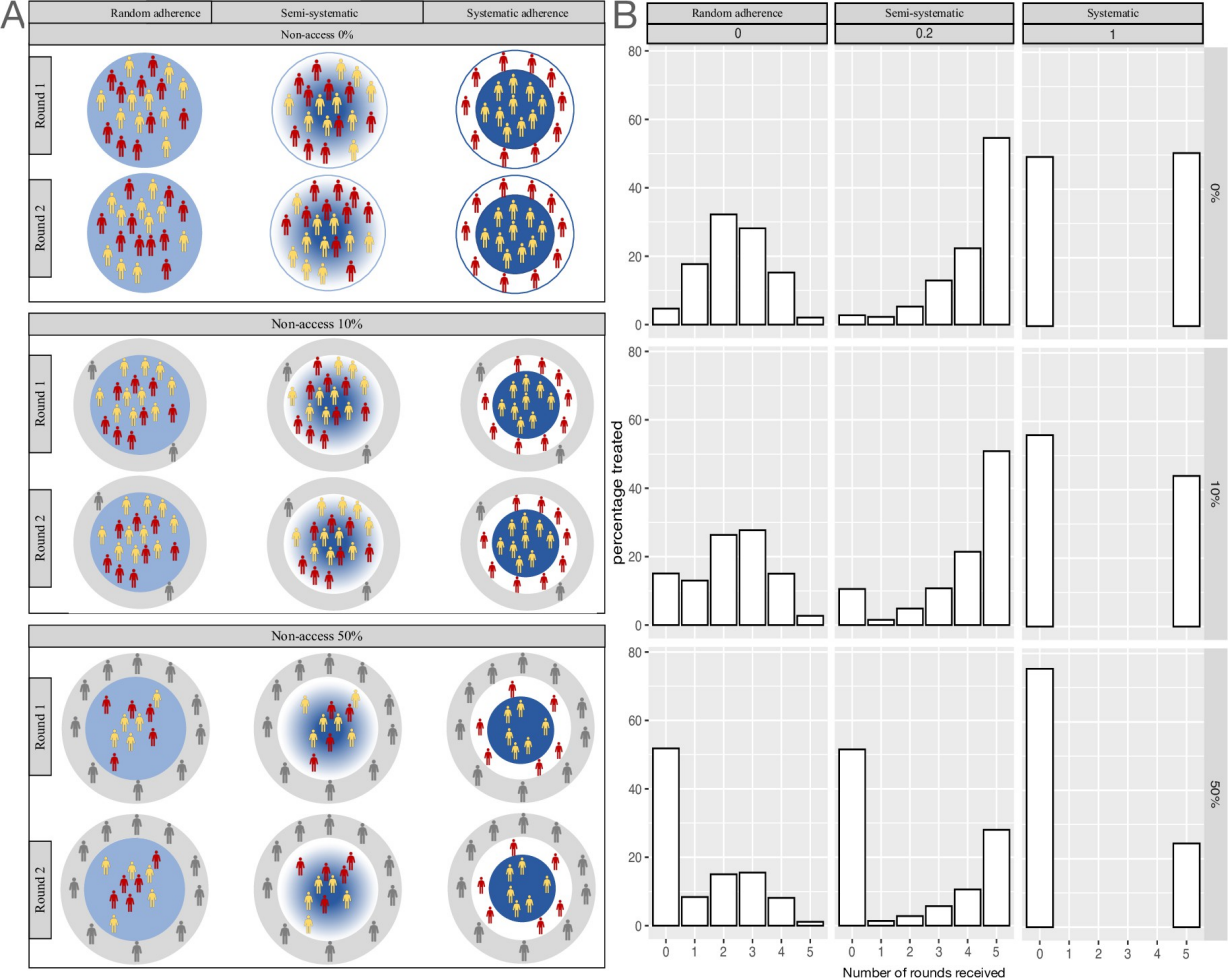
Model schematic. A. Adapted from Dyson et al [14] visualising how varying degrees of adherence to treatment from 0 to 1 impact treatment participation over successive rounds. The background colour represents the probability that a person will participate in MDA from white (no participation) to dark blue (definitely participating). We augment these with population partitioning into those who have access to treatment (those in white or blue) and those with no access to treatment (grey). B. The proportion of the population that have received 1, 2, 3, 4, or 5 rounds of treatment after five years as a function of example values of adherence (0, 0.2 and 1) and non-access (0%, 10% and 50%) experiencing 85% target coverage to the with-access population. Human outlines attributed to Microsoft clipart.

We partitioned all populations into two groups; access and non-access. Those in the non-access group did not receive treatment and were not included in relevant prevalence estimates. The access group were the target of the 65% coverage. This allocation occurs when each individual is born, and is generated from a binomial distribution parameterised for a Bernoulli draw (i.e., N trials = 1). This allocation is static for the duration of the simulation. This partitioning of the population is akin to adherence in previous work on lymphatic filariasis [29] onchocerciasis [30], human African trypanosomiasis [31] or hookworm [32]. Additionally, we assume homogenous mixing between individuals and vectors, regardless of their treatment access, such that those with no access still contribute to transmission (Fig 1) and therefore the overall infection dynamics of a community. The implications of this assumption are addressed in the discussion.

To account for treatment adherence, each individual ≥five-years-old in the treatment access group was assigned a probability of treatment. The group excluded from treatment and surveillance have 0 (zero) probability of being treated. For those with access to treatment, the probability of treatment is a function of the overall target treatment coverage and the correlation in adherence to treatment across rounds, as derived by Dyson et al [14]. In short, as shown in Fig 1A, when there is a weak correlation in treatment participation between rounds (systematically low adherence), treatment participation across the population is more randomly distributed, such that eventually, over successive rounds everyone is likely to be treated at some point. Alternatively, when the correlation between who gets treated from one round to the next is high, adherence becomes systematic, such that whether someone participates in treatment is determined by their behaviour in the first round–if they did not participate in round one, they will not participate in any subsequent rounds. The more systematic treatment adherence is, the more individuals there are that participate in all five rounds of treatment, but also the more individuals there are that are rarely–if ever–treated (Fig 1B). However, as more people are excluded from treatment with declining access the proportion of the total population who receive any treatment at all declines (Fig 1B), such that the effective overall coverage (% of the population) is lower. This is most notable when adherence is systematic.

With the adaptations to this model, we investigated the probability that the normal routine of MDA would result in control (reaching EPHP) given the population observed and extent of non-access. We then investigated how many rounds would be necessary to reach EPHP. Finally, to observe infection dynamics when treatment programmes cease, we next selected populations that had reached the EPHP indicator for each surveillance group, by year five when surveillance most often occurs.

## Results

### *Impact* of exclusion on reaching EPHP targets

**Fig 2:**
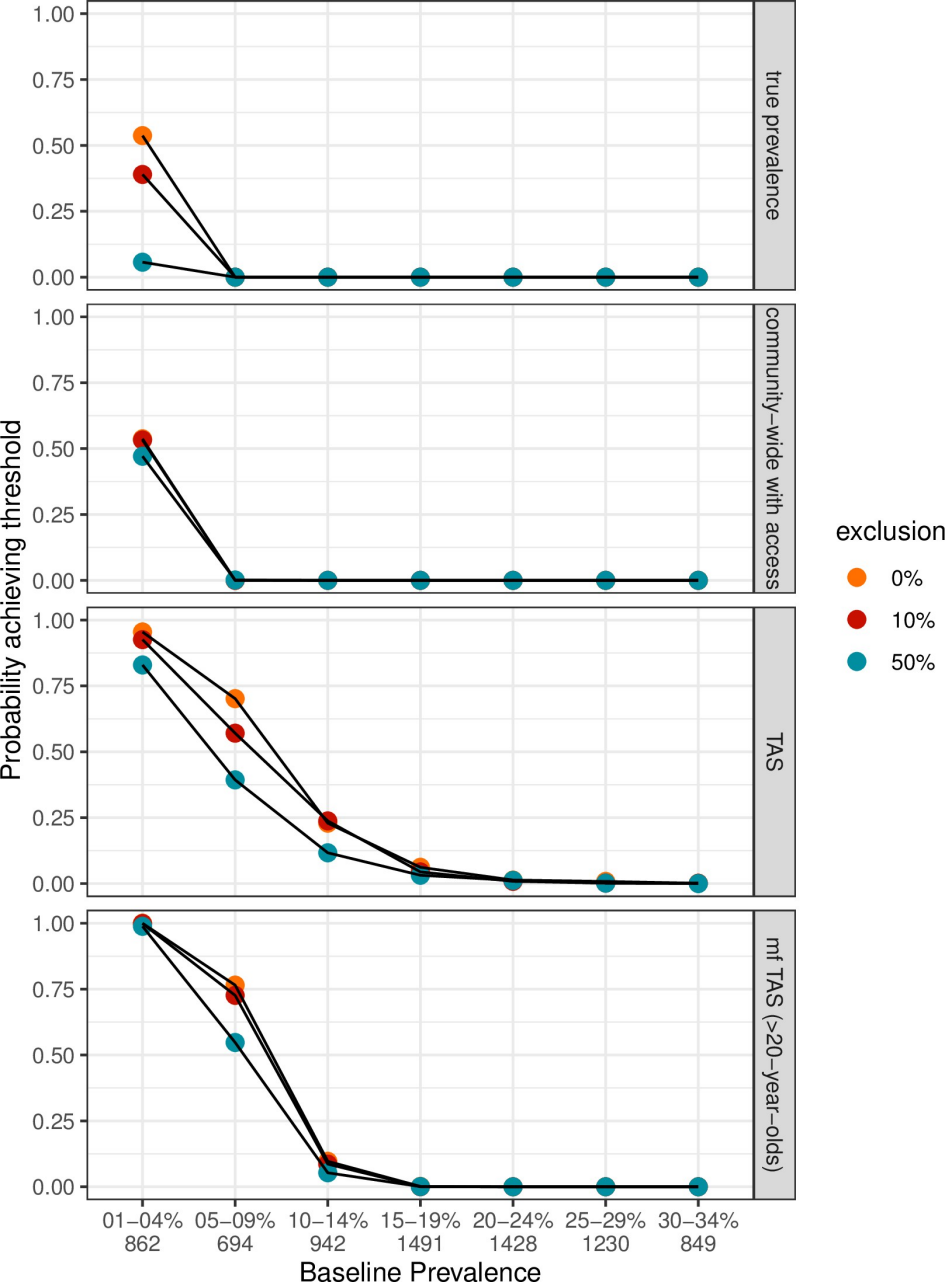
The probability of achieving elimination as a public health problem after five years of treatment as a function of exclusion and the baseline prevalence. The number of populations in each baseline prevalence group is shown on the x-axis. The x axis is the true prevalence in the whole population >five-years-old. The target for all panels apart from the mf TAS is ≤1% prevalence, whilst the target for mf TAS was ≤2%. Exclusion is shown in orange for 0% exclusion, red for 10% exclusion, and blue for 50% exclusion. We assume a moderate correlation value for adherence to treatment of 0.2 [14].

We observed the probability of achieving elimination as a public health problem after six years of treatment, as a function of the baseline true prevalence and the proportion excluded from treatment and surveillance (Fig 2). The number of populations in each baseline prevalence bin are shown in Fig 2. A total of 14,145 of the simulated populations were used, however we capped Fig 2 observations at 34% prevalence as per data from previous work [23].

There are three main observations; first, exclusion reduces the probability of reaching EPHP even in very low prevalence locations (Fig 2 True Prevalence). With a starting prevalence of 1–5%, five years of MDA yields just over a 50% probability of achieving EPHP when treatment is provided as intended to a whole community, with everyone who is treated also participating in surveillance–essentially whether 65% coverage will achieve EPHP is as unlikely as it is likely. This reduces significantly with increasing levels of non-access (Fig 2, True Prevalence red and blue versus orange).

Secondly, the perceived probability of achieving EPHP is driven in part by the population in which you look. This is most pronounced in lower prevalence communities as infections are easier to miss. Increasingly narrowed observed groups overestimate the probability of reaching targets (comparing between panels Fig 2). For example, community-wide surveillance in those with access to treatment, even in the presence of exclusion captures that five years of treatment will not reach EPHP when prevalence is >5%, whilst observations in only those >20-years-old suggests ~ 10% chance in the same baseline prevalence populations. This difference is exaggerated in the TAS observation, where EPHP appears possible to achieve even in populations where prevalence is >10%.

This suggests, as our final observation, that regardless of the level of exclusion, TAS does not reflect the dynamics of the wider population (Fig 2, True Prevalence vs TAS panels), reaching EPHP indicator targets despite not actually having done so in the wider population. There is a significantly higher probability of appearing to have achieved the EPHP threshold using TAS with FTS in six- and seven-year-olds. This is particularly evident the lower the prevalence. With no exclusion, there appears to be a 25% probability that the target will be (perceived to be) reached in populations with a true starting prevalence of 10–14%. However, in all other observation groups, regardless of exclusions to treatment, by 10–14% prevalence, the baseline prevalence is too high to control infection in five years even with full access to treatment.

**Fig 3:**
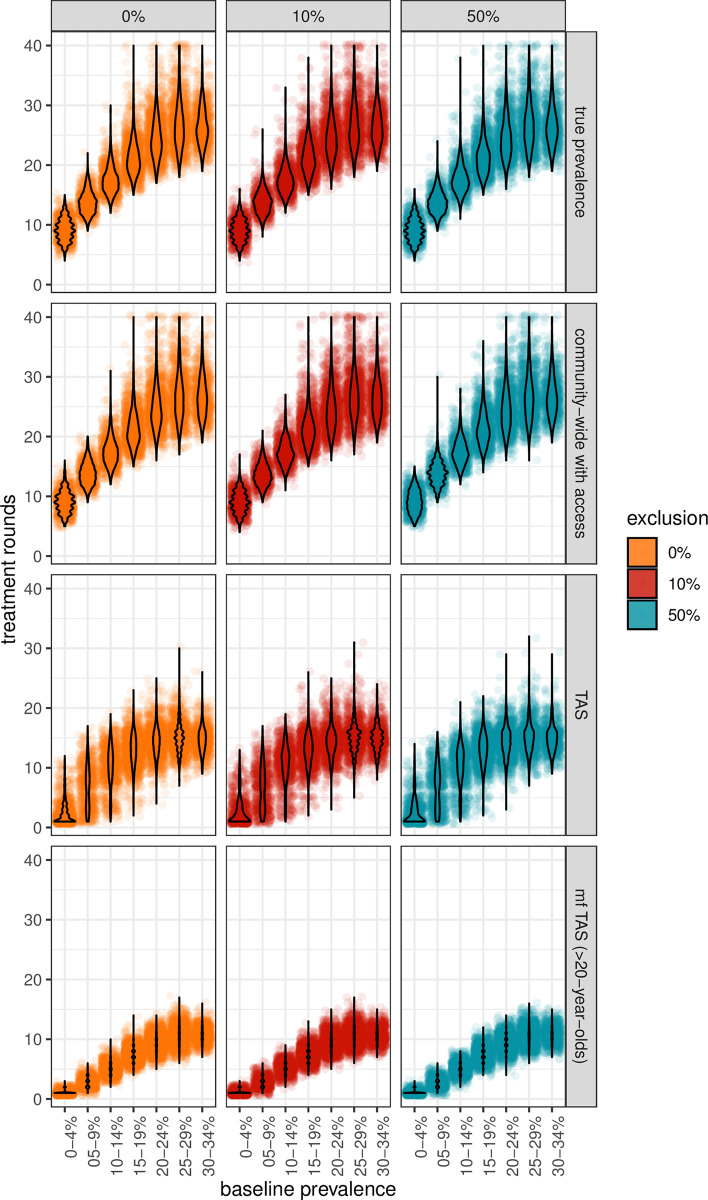
The number of treatment rounds needed to reach the elimination as a public health problem target (1% for the mf TAS and 2% for everything else). This has been capped at 20 years of treatment.

We next investigated the number of rounds of treatment needed to reach the appropriate elimination targets for each surveillance group as a function of exclusion. It was hoped that by cross referencing the true baseline prevalence with the number of years of treatment necessary to reach the EPHP target, and exclusion, this could provide an indicator of the true baseline prevalence and the level of exclusion present in a population when treatment had been ongoing for more years than was projected to be necessary. However, if surveillance is occurring only in the group being treated it is not possible to detect the presence of excluded groups (Fig 3). The group being observed continues to have a strong impact on the dynamics observed. For example, in Fig 3 the >20-year-old group estimates that populations with prevalence up to 10% could be largely controlled within five years, whilst in the true prevalence observations even 10% prevalence would require a minimum of five years up to 15–20 years to control with MDA. Similarly, the mean behaviour of the TAS routine suggests control would be possible even at the highest prevalences on average within 10–15 years, whilst the true observations suggest this is more like 25 years. The higher the prevalence the more uncertain the number of years necessary to control infection.

### Impact of exclusion on maintaining EPHP

**Fig 4:**
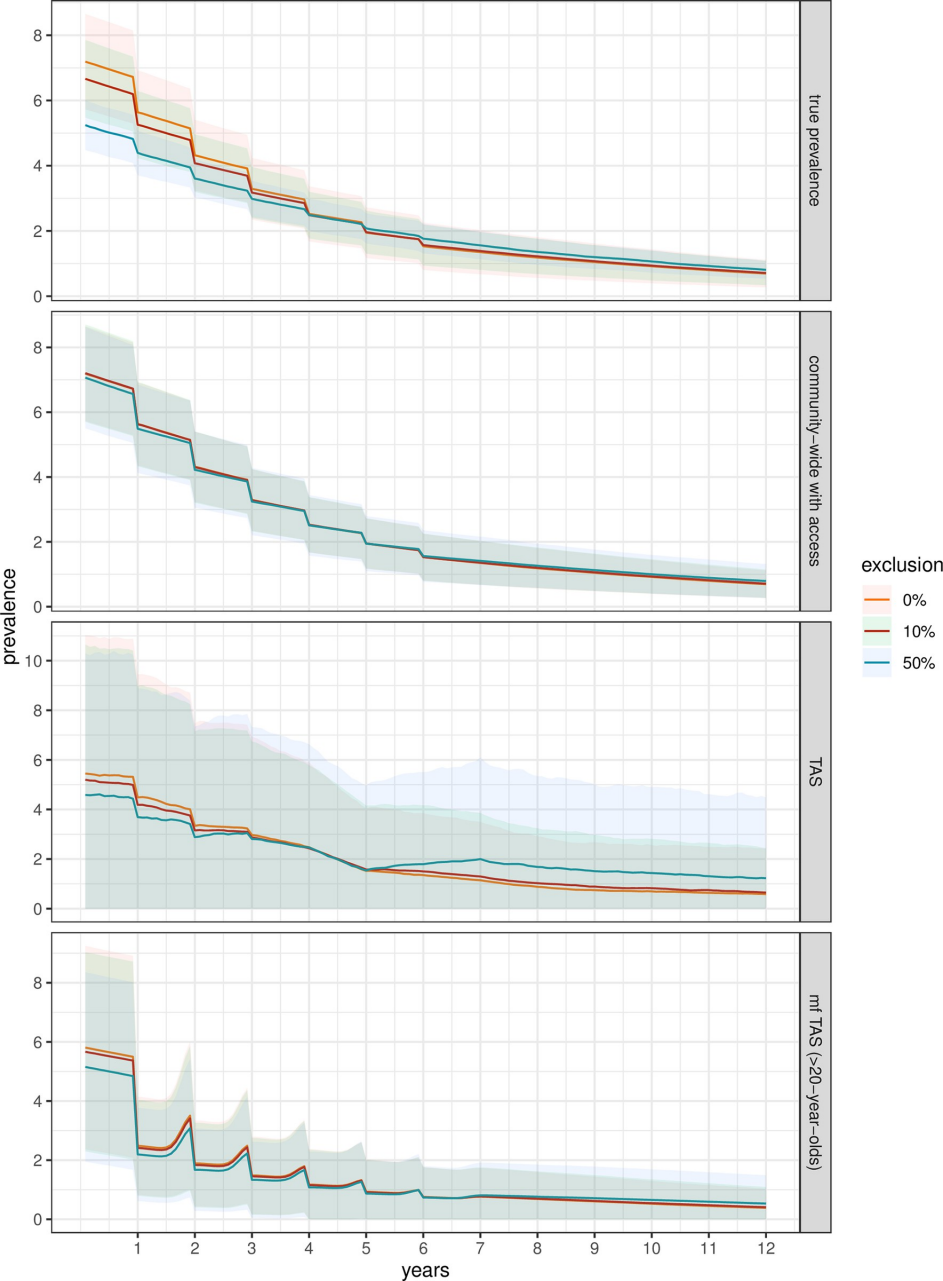
Prevalence over 12 years with standard deviation of the mean observed in four surveillance groups as a function of treatment and surveillance exclusion. True prevalence refers to prevalence in the whole population ≥five-years-old. Community-wide with access is also the population ≥five-years-old but only in those with access to treatment and surveillance. TAS simulates the observations made in six- and-seven-year-olds with access to treatment. Finally, the mf TAS quantifies prevalence by detectable mf in >20-year-olds. Exclusion is represented in orange for 0%, red with green standard deviation for 10% and blue for 50%. As described in the methods, treatment is given for five years plus one likely given due to the timing and the way treatment is ordered and distributed. The populations used in these simulations had reached elimination as a public health problem after five years of treatment. Note the different limits on the y axes.

Finally, we observed the infection dynamics across the four observed surveillance groups as a function of the excluded proportion of the population. Based on FTS, infection prevalence was observed as: 1) true prevalence in the whole population aged ≥five-years-old 2) measured prevalence in everyone over five-years-old with access to treatment and therefore included in surveillance 3) as observed during the TAS, in six- and-seven-year-olds with access to treatment. Prevalence quantified from detectable mf was used in group 4) in over 20-year-olds with access to treatment and included in surveillance. This is shown for 0%, 10%, and 50% exclusion. The number of populations used for each simulation (i.e., that were used to generate each line of Fig 4) is shown in Table B in S1 Supplementary Material.

Here, we make two observations; first, the extent of exclusion has little impact on prevalence in those populations that do reach EPHP, and the maintenance of EPHP once treatment stops (Fig 4), though the prevalence is indeed lower in populations that have 50% exclusion and still successfully reach EPHP. To less of a degree this is also the case for those with 10% exclusion (Fig 4 True Prevalence panel).

Secondly, there is little difference between exclusion groups in the overall mean behaviour of the dynamics, however there is a distinct increase in variation around the mean dynamics when observing infection with standard TAS methods, which is most pronounced the 50% exclusion group after treatment stops. Again, this suggests that often, the TAS method will not reflect true population dynamics.

## Discussion

In this modelling investigation we illustrated how the correlation between treatment access and surveillance exclusion can impact the success of, and the M&E for, intervention programmes. A repeated lack of access to treatment and exclusion from surveillance impedes the probability that a population will reach the EPHP target after five years of treatment, but it does not impact the maintenance of it in those populations that do reach the target and subsequently stop MDA. Five years of treatment is not sufficient to control the disease in most cases; even when treatment is available to 65% of the eligible population and surveillance captures all the infections in the treated population, there is only a 50:50 chance the MDA programme will succeed. In addition, the TAS is not an appropriate tool for quantifying population-level dynamics. Treatment of, and observations in only six-and-seven-year-olds does not control infection and overestimates the probability of MDA success, with significantly more variation around the mean dynamics in the results obtained from the TAS, which increases with increasing exclusion. Across all these observations it is also evident that the observation group has a strong impact on the estimated dynamics. Intuitively, the more constrained the group under observation, the less the observed dynamics reflect that of the wider population. Crucially, we show that excluded groups cannot be detected when observations are made only in the surveillance group with access to treatment. As MDA is the cornerstone of control for five of the WHO NTDs, all with ambitious prevalence targets set for 2030, our results highlight the need for accurate coverage estimates and the importance of accessing demographic groups that may be easily overlooked in treatment and surveillance efforts.

When programmes are evaluating their progress, the discrepancies sometimes seen between coverage survey numbers and treatment numbers can indicate when those conducting surveys and those distributing treatment have access to different individuals, groups, or communities [33], suggesting groups with non-access. This is driving a larger discussion about how to identify, measure and engage with this group of untreated individuals [3,34]. Our analysis highlights the importance of designing surveys that aim to minimise bias. By identifying the people that make up these discrepancies, we are potentially also more likely to increase the uptake of MDA treatments. For example, it has been noted that there is a distinct intersection between NTD treatment access and disabilities, particularly when NTDs like filariasis or leprosy cause stigmatising morbidity and disabilities, impacting treatment reach [7]. There is also evidence that access to treatment for one disease is correlative to healthcare access more broadly, including other treatment programmes like malaria [35], meaning if a person has no access to treatment for one disease, it is likely they do not have access to many treatments. Additionally, whilst evidence suggests that women are less adherent to treatment, men are often outside of the home at the time of MDA and at the time of CES and surveillance [21].

**Fig 5:**
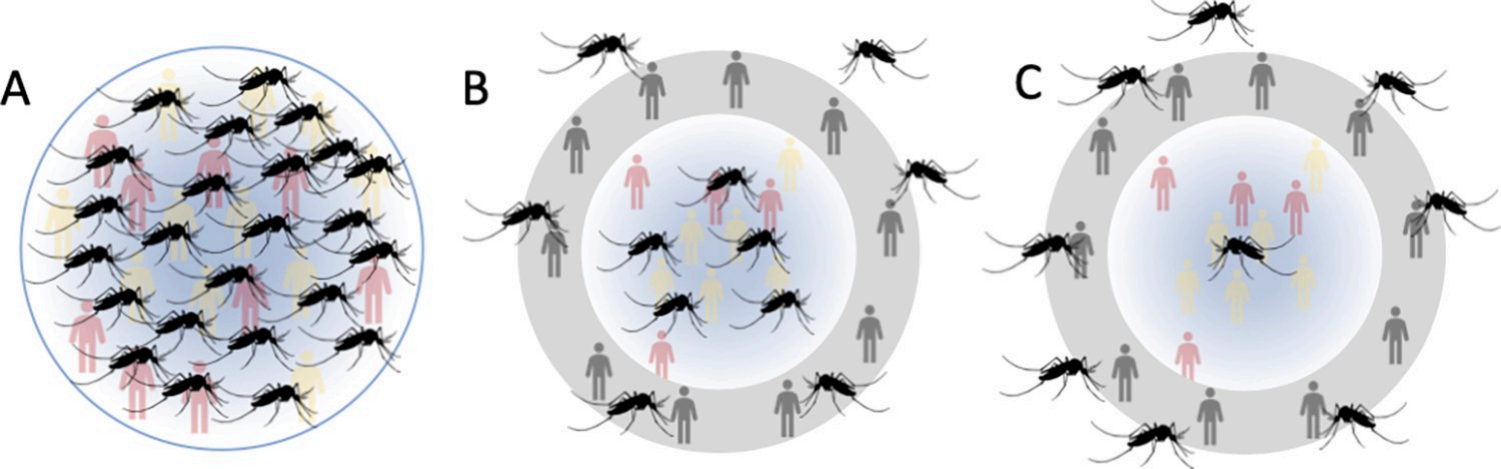
Distribution of mosquito vectors across three theoretical scenarios. A. When the whole population has access to treatment and are included in surveillance, with participation controlled by adherence. Vectors are also distributed evenly across the population, so everyone has the same risk of being bitten/ infected. B. The population is separated based on who has access to treatment and is included in surveillance, but those excluded are not at any higher risk of contracting disease. C. Those excluded are also more likely to be bitten/ infected therefore harbouring more disease than in the group with access to treatment, who are less likely to be bitten. Human outlines attributed to Microsoft.

There are a number of limitations and simplifying assumptions in this analysis. In the absence of non-access, the model assigns a bite risk to each individual, drawn from a population-level gamma distribution (Fig 5A). As a function of the way in which the population is separated into those with access to treatment and those without, we maintain the assumption that everyone, regardless of treatment access, has a bite risk drawn from this same distribution (Fig 5B). However, it is possible that the effects we have seen here would be exaggerated if the non-access group were also at a higher risk of being bitten (Fig 5C). There are numerous reasons for and quantifications of the heterogeneities in bite risk for lymphatic filariasis, which have been compared within [36] and between locations [37]. It is therefore feasible that for the same reason treatment is not provided, an individual may be at higher risk of being bitten—for example due to employment, home location, poor bed net usage or lack of screened windows. Such a correlation between adherence and bite risk has been shown to significantly impact the probability of reaching EPHP for lymphatic filariasis [23]. Furthermore, the correlation between treatment access and lack of surveillance may be transient depending on the time delay between the two events.

We also make the assumption that all human hosts are homogeneously mixing with the vectors regardless of treatment access and inclusion in surveillance. The assumption of homogeneous mixing is common, however there is evidence that accounting for non-homogenous mixing can explain maintained infection prevalence [38]. Whilst it is likely that a non-homogenous mixing assumption would better describe the behaviour within the population, the point still stands, that increasing disparities in treatment access could negatively impact treatment programme success. We also note, that as these are generic, stylised simulations, the population age structure may be different to that of a location where lymphatic filariasis may be more common, where often the population is skewed towards a greater number of children and fewer adults. This will impact observations made in the six- and seven-year-old group, where a smaller group is likely more prone to random extinctions and resurgences that do not reflect the dynamics of the wider population. Finally, here we used infection prevalence as a metric for long term intervention success. It is possible that infection intensity will have reduced more significantly. However, there is ongoing debate around the relationship between morbidity from infection and infection intensity quantified by diagnostics that lack sensitivity or are hard to use. Given that the public health targets focus on prevalence we have done so here also but suggest further work can be done to explore insights from infection intensity.

To conclude, we show that exclusion to treatment and from surveillance impacts the probability of reaching public health targets, but in the small proportion of populations that do reach the threshold, it does not impact maintenance. The TAS is an insufficient tool for quantifying population-level dynamics, and finally, it is not possible to identify the presence of exclusion, when observations only occur in the same group being treated. Whilst we did not investigate this here, it may be that indirect measures such as xenomonitoring may provide indirect signals. Adapting models such as this one to inform on this is a valuable next step towards identifying and quantifying groups with limited access to treatment, to better inform intervention programmes and end-game management.

## Supporting information

S1 Supplementary material Table A. Parameter values used for the simulations. Fig A. Distribution of prevalences across the 14,145 simulated populations. Fig B. The relationship between the vector-to-host ratios, k (bite aggregation parameter) and prevalence. Increasing vector-to-host ratio is generally accompanied by a more random distribution of bites–i.e., a larger value of k as the distribution of bites becomes more randomly (Poisson) distributed in the population. Table B. The number of populations used in Fig 4 for each combination of observation group and exclusion proportion (i.e., each line in each figure is represented by a row in the below table). These are the numbers of populations under each scenario that appeared to have reached EPHP after 5 years of treatment. Fig C. Systematic non-access on the y-axis and treatment adherence correlation on the x-axis. These are figures from previous analyses conducted on high and low transmission populations (high *=* starting prevalence ~ 23% and low ~5%). The deepening colour relates to a decreasing percentage point difference between observed and true infection prevalence in the two settings.
(DOCX)
