# Supplementary material for: How correlations between treatment access and surveillance inclusion impact neglected tropical disease monitoring and evaluation—A simulated study
Source: PLoS Negl Trop Dis. 2023 Sep 6;17(9):e0011582. doi: 10.1371/journal.pntd.0011582 (PMC10506705; doi:10.1371/journal.pntd.0011582)
Supplement: S1 Supplementary material — Table A. Parameter values used for the simulations. Fig A. Distribution of prevalences across the 14,145 simulated populations. Fig B. The relationship between the vector-to-host ratios, k (bite aggregation parameter) and prevalence. Increasing vector-to-host ratio is generally accompanied by a more random distribution of bites–i.e., a larger value of k as the distribution of bites becomes more randomly (Poisson) distributed in the population. Table B. The number of populations used in Fig 4 for each combination of observation group and exclusion proportion (i.e., each line in each figure is represented by a row in the below table). These are the numbers of populations under each scenario that appeared to have reached EPHP after 5 years of treatment. Fig C. Systematic non-access on the y-axis and treatment adherence correlation on the x-axis. These are figures from previous analyses conducted on high and low transmission populations (high = starting prevalence ~ 23% and low ~5%). The deepening colour relates to a decreasing percentage point difference between observed and true infection prevalence in the two settings. (DOCX) [file pntd.0011582.s001.docx]

**How correlations between treatment access and surveillance inclusion**

**impact neglected tropical disease monitoring and evaluation – a modelling investigation.**

Jessica Clark*^1,2^, Emma L. Davis^2^, Joaquin M. Prada^3^,

Katherine Gass^4^, Alison Krentel^5,6^, T. Déirdre Hollingsworth^2^

1. School of Biodiversity, One Health & Veterinary Medicine, University of Glasgow, Glasgow, Scotland
2. Big Data Institute, Neglected Disease Modelling Consortium, University of Oxford, Oxford, England
3. School of Veterinary Medicine, [Faculty of Health and Medical Sciences](https://www.surrey.ac.uk/faculty-health-medical-sciences), University of Surrey, Guildford, England
4. Neglected Tropical Diseases Support Center, Task Force for Global Health, Decatur, Georgia
5. School of Epidemiology and Public Health, University of Ottawa, Ottawa, Canada
6. Bruyère Research Institute, Ottawa, Canada

This supplementary material accompanies the manuscript titled above. All relevant code can be found at <https://github.com/iamjessclark/non_access.git>

Table A. Parameter values used for the simulations.

| **Definition** | **Value** | **Source** |
| --- | --- | --- |
| Human Host | | |
| Population sizes | ~1500 | NA |
| Host death rate | 0.00167/ month | NA |
| Bite risk overdispersion | [0.01 - 0.1] | (1, 2) |
| Vector | | |
| Bite rate | 10/ mosquito/month | (3) |
| Mosquito infection proportion | 0.37 | (4, 5) |
| L3 uptake and development | 4.395 | (4, 6) |
| Mosquito death rate | 5/ month | (7) |
| Vector-to-host ratio group 1 | [1 – 5] | NA |
| Vector-to-host ratio group 2 | [10 – 15] | NA |
| Vector-to-host ratio group 3 | [15 – 25] | NA |
| Vector-to-host ratio group 4 | [25 – 30] | NA |
| Vector-to-host ratio group 5 | [30 – 40] | NA |
| Bite risk aggregation group 1 | 0.03 | (6) |
| Bite risk aggregation group 2 | 0.16 | (6) |
| Bite risk aggregation group 3 | 0.27 | (6) |
| Bite risk aggregation group 4 | 0.37 | (6) |
| Worm & microfilariae | | |
| Bite risk aggregation group 5 | 0.48 | (6) |
| MF birth rate | 1/ per female worm | (3) |
| Proportion L3 leaving mosquito | 0.414/bite | (8) |
| Proportion L3 leaving mosquito and entering host | 0.32 | (7) |
| Proportion L3 entering host and developing into adults | 0.00275 | (9, 10) |
| Adult worm death rate | 0.1 | (11) |
| Intervention (Ivermectin & Albendazole) | | |
| Target coverage | 65% | (12) |
| Systematic non-adherence | [0 – 1] | (13) |
| Duration of fecundity reduction | 6 months | (14) |
| Systematic exclusion | 0% - 50% | NA |
| MF killed | 0.99 | (14) |
| Worms killed | 0.35 |  |

*Figure A. Distribution of prevalences across the 14,145 simulated populations.*

**** Figure B. The relationship between the vector-to-host ratios, k (bite aggregation parameter) and prevalence. Increasing vector-to-host ratio is generally accompanied by a more random distribution of bites – i.e., a larger value of k as the distribution of bites becomes more randomly (Poisson) distributed in the population.

| observed | exclusion | count |
| --- | --- | --- |
| TAS | 0 | 1648 |
| TAS | 0.1 | 1499 |
| TAS | 0.5 | 1164 |
| community-wide with access | 0 | 463 |
| community-wide with access | 0.1 | 459 |
| community-wide with access | 0.5 | 407 |
| mf TAS (>20-year-olds) | 0 | 1486 |
| mf TAS (>20-year-olds) | 0.1 | 1446 |
| mf TAS (>20-year-olds) | 0.5 | 1283 |
| true prevalence | 0 | 463 |
| true prevalence | 0.1 | 336 |
| true prevalence | 0.5 | 49 |

Table B. The number of populations used in Figure 4 for each combination of observation group and exclusion proportion (i.e., each line in each figure is represented by a row in the below table). These are the numbers of populations under each scenario that appeared to have reached EPHP after 5 years of treatment.

**Adherence versus access.**

By visualising the percentage point differences between observed and true infection prevalence as a function of interacting adherence and access values in both the high baseline and low baseline populations after five years of treatment, we show that treatment access has the strongest impact on infection prevalence (Figure A, where the lighter the colour the higher the percentage point difference). This is most notable for the high prevalence location, where there is essentially more room for these greater disparities in prevalence (Figure A).

Figure C. Systematic non-access on the y-axis and treatment adherence correlation on the x-axis. These are figures from previous analyses conducted on high and low transmission populations (high = starting prevalence ~ 23% and low ~5%). The deepening colour relates to a decreasing percentage point difference between observed and true infection prevalence in the two settings.

1. Das PK, Manoharan A, Subramanian S, Ramaiah KD, Pani SP, Rajavel AR, et al. Bancroftian filariasis in Pondicherry, south India--epidemiological impact of recovery of the vector population. Epidemiological Infection. 1992;108(3):483-93.10.1017/s0950268800049992

2. Subramanian S, Pani SP, Das PK, Rajagopalan PK. Bancroftian filariasis in Pondicherry, South India: 2. Epidemiological evaluation of the effect of vector control. Epidemiological Infection. 1989;103:693-702

3. Irvine MA, Stolk WA, Smith ME, Subramanian S, Singh BK, Weil GJ, et al. Effectiveness of a triple-drug regimen for global elimination of lymphatic filariasis: a modelling study. The Lancet Infectious Diseases. 2017;17(4):451-8.10.1016/s1473-3099(16)30467-4

4. Gambhir M, Michael E. Complex ecological dynamics and eradicability of the vector borne macroparasitic disease, lymphatic filariasis. PLoS One. 2008;3(8):e2874.10.1371/journal.pone.0002874

5. Subramanian S, Krishnamoorthy K, Ramaiah KD, Habbema JD, Das PK, Plaisier AP. The relationship between microfilarial load in the human host and uptake and development of Wuchereria bancrofti microfilariae by Culex quinquefasciatus: a study under natural conditions. Parasitology. 1998;116 ( Pt 3):243-55.10.1017/s0031182097002254

6. Irvine MA, Reimer LJ, Njenga SM, Gunawardena S, Kelly-Hope L, Bockarie M, et al. Modelling strategies to break transmission of lymphatic filariasis--aggregation, adherence and vector competence greatly alter elimination. Parasites & Vectors. 2015;8:547.10.1186/s13071-015-1152-3

7. Ho BC, Ewert A. Experimental transmission of filarial larvae in relation to feeding behaviour of the mosquito vectors. Transactions of The Royal Society of Tropical Medicine and Hygiene. 1967;61(5):663-6

8. Hairston NG, de Meillon B. On the inefficiency of transmission of Wuchereria bancrofti from mosquito to human host. Bulletin of the World Health Organization. 1968;38:935-41

9. Stolk WA, de Vlas SJ, Borsboom GJ, Habbema JD. LYMFASIM, a simulation model for predicting the impact of lymphatic filariasis control: quantification for African villages. Parasitology. 2008;135(13):1583-98.10.1017/S0031182008000437

10. Norman RA, Chan MS, Srividya A, Pani SP, Ramaiah KD, Vanamail P, et al. EPIFIL: The development of an age-structured model for describing the transmission dynamics and control of lymphatic filariasis. Epidemiological Infection. 2000;124:529-41

11. Evans D, Gelband H, Vlassoff C. Social and economic factors and the control of lymphatic filariasis: a review. Acta Tropica. 1993;53:1-26

12. World Health Organization. Ending the neglect to attain the sustainable development goals. Geneva: World Health Organization; 2020.

13. Dyson L, Stolk WA, Farrell SH, Hollingsworth TD. Measuring and modelling the effects of systematic non-adherence to mass drug administration. Epidemics. 2017;18:56-66.10.1016/j.epidem.2017.02.002

14. Ismail MM, Jayakody RL, Weil GJ, Nirmalan N, Jayasinghem KSA, Abeyewickrema W, et al. Efficacy of single dose combinations of albendazole, ivermectin and diethylcarbamazine for the treatment of bancroftian filariasis. Transactions of the Royal Society of Tropical Medicine and Hygiene. 1998;92:94-7
